# Hypermethylation of *DcR1* Gene-based Biomarker in Non-invasive Cancer Screening of Vietnamese Cervical Cancer Patients

**Published:** 2018-03

**Authors:** Phuong Kim TRUONG, Thuan Duc LAO, Thuy Ai Huyen LE

**Affiliations:** 1. Dept. of Molecular and Environmental Biotechnology, Faculty of Biology and Biotechnology, University of Science, Vietnam National University, Ho Chi Minh, Vietnam; 2. Dept. of Pharmaceutical and Medical Biotechnology, Faculty of Biotechnology, Ho Chi Minh City Open University, Ho Chi Minh, Vietnam

**Keywords:** Cervical cancer, *DcR1*, Hypermethylation, MSP, Vietnamese population

## Abstract

**Background:**

The infection of human papillomavirus (HPV) has been considered as the common cause of cervical cancer, which is the leading cause of cancer death in women, in Vietnam. Recently, hypermethylation at tumor suppressor genes (TSGs) has been also demonstrated to be an early epigenetic event and cofactor in human cancer, including cancer of cervix. This study evaluated the frequency of *DcR1* gene promoter hyper-methylation status as well as whether did or not an association between patterns of DNA hypermethylation and high-risk HPV infection, led to risk of cervical cancer.

**Methods:**

Methylation-Specific-PCR (MSP) was performed to analyze hypermethylation status from 109 liquid-based Papanicolaou test samples, archived and admitted from the Medic Medical Center and Au Lac Clinic Laboratory, Vietnam, from 2011–2014, a kind of non-invasive samples identified whether HPV/or non-HPV, high-risk/low-risk HPV infection.

**Results:**

*DcR1* promoter was differentially methylated in 50% cases of high-risk HPV genotype 16 and 18 infected samples. In contrast, a low frequency of hypermethylated DcR1 promoter was found in low risk HPV genotype infected sample (16.0%), and non-HPV infected sample (14.6%). A trend toward positive association was found between hypermethylation of DcR1 gene and HPV exposure was observed (*P*=0.0005). Moreover, the odds ratio (OR) and relative risk (RR) were found in statistical significant value (OR=5.63 (95%CI = 2.25 – 14.07, *P*<0.01), RR=3.31 (95%CI = 1.75 – 6.26, *P*<0.01)).

**Conclusion:**

The hypermethylation of *DcR*1 gene promoter is a significant characteristic of high-risk HPV infected samples in Vietnamese cervical patients. The OR and RR values showed that the strong correlation between *DcR1* hypermethylation and high-risk HPV infection, in which increased the risk of cervical cancer. The combination of *DcR1* hypermethylation and HPV detection based biomarker could be used in noninvasive samples obtained from high-risk cancer patients, offer significant practical advantages.

## Introduction

DcR1 (Decoy receptor 1), also known as TNFRSF10C (Tumor necrosis factor receptor superfamily member 10C), locates on 8p21.3, is homologous of Fas and tumor necrosis factor receptor 1 (TNFR1) trigger apoptosis upon engagement by their cognate death ligands. DcR1 receptor contains an extracellular TRAIL-binding domain and a transmembrane domain, while completely lacks the intracellular death domain, hence is not capable of inducing apoptosis. Additionally, DcR1 has been found to be a p53-regulated DNA damage-inducible gene. The specific sensitivity of cancer cells to the TRIAL apoptosis-inducing activity may be interpreted by the observation of DcR1 expression in many non-cancerous cells (1). The loss expression of DcR1 by aberrant methylation have been reported in many human tumors, like lung, colon, prostate, glioblastoma, head and neck cancer, etc. (2, 3). There is not much data about *DcR1* promoter methylation in cervical cancer, especially, in Vietnamese cervical cancer patients. Thus, in order to determine whether promoter hypermethylation profile in Vietnam, the data on *DcR1* hypermethylation was evaluated and investigated in a series of clinical HPV infected samples collected from Vietnamese patients were enrolled in current study to develop method for prognosis and early diagnosis of cervical cancer based on the detection of *DcR1* methylation status.

In Vietnam, cervical cancer is the leading cause of cancer death in women. The high prevalence of cervical cancer cases was observed in reached to 527624 cases (ASR = 14.0/100000) and deaths were 265672 cases (ASR = 6.8/100000) (4). The etiology of cervical cancer has been reported to be associated with several types of human papillomavirus (HPV). Especially, the common high-risk genotypes of HPV are HPV-16 and -18, identified as being key roles in the majority of cervical cancer, counting for approximately 70% (5–8). In Vietnam, the prevalence of high-risk HPV infection was ranged from 24.5% to 56.8%. Meanwhile, the prevalence of cervical infection within HPV type 16 and/or HPV type 18 was from 3.1% to 7.4% (9). Besides the viral infection, cervical cancer progression is a multi-steps process accumulating of genetic and epigenetic alterations in regulatory genes, leading to the inactivation or loss of expression of tumor suppressor genes (TSGs) or activation of oncogenes combined with the high-risk HPV infection and integration (5, 10, 11). DNA methylation, reported to play a key role in gene silencing, also is demonstrated in cervical tumorigenesis (10, 11). Here, the aim was to evaluate the frequency of hypermethylation of CpG belonged to the promoter of *DcR1* gene, in Vietnamese population, as well as, to study about the association between the epigenetic event, hypermethylation, and high-risk HPV infection leading to the cancer of cervix. Notably, the kind of non-invasive materials, such as liquid-based Pap’s test specimens (PAP), had more advantages and easily developed for non-invasive method applied in Vietnamese cervical cancer patients’ early diagnosis and prognosis based on DNA methylation-specific PCR (12, 13).

## Materials and Methods

### Ethics statement

We used anonymized routine specimen surplus to clinical requirements for assay validation, adhering to a governance framework agreed by and with a Medic Medical Center and Au Lac Clinic Laboratory ethics agreement relating to the use of specimen surplus to clinical needs.

### Clinical sample collection

Overall, 109 liquid-based Pap test samples were archived and admitted from the Au Lac Clinic, Vietnam, from 2011 – 2014. For input confirmed, the detection of HPV was carried out by using LightPoweriVA HPV genotype PCR-RDB Kit (Code: VA. A02-003E, Viet-A Corporation, Vietnam). All samples were divided into two groups: negative HPV infection group, which consisted of 48 samples; and positive HPV infection group, in which composed of 36 high-risk HPV (HPV genotype 16, 18 and other high-risk genotypes) infected samples and 25 low-risk HPV infected samples.

### DNA isolation, bisulfite modification, MSP and BSP assay

Total of genomic DNA was isolated from PAP samples by phenol/chloroform method. Then, DNA concentration of DNA was quantified by the absorbance at OD_260_ and OD_280_. The pure preparation of DNA with OD_260_/OD_280_ ratio values of 1.8 to 2.0 was used to the bisulfite DNA modification assay. The bisulfite modification was carried out with approximately 2 μg genomic DNA of each sample by DNA modification Kit (Epitect Kit, Qiagen). The final precipitate was eluted in a volume of 20 μl for MSP assay. Two pairs of primer were used to amplify the regions of interest. One pair recognized a sequence in which CpG sites were methylated (unmodified by bisulfite treatment). Other pair recognized a sequence in which CpG sites were unmethylated (modified to UpG treatment). The primer sequences and X°C for each annealing temperatures were noted in Table 1.

**Table 1:** Methylated and unmethylated of primer *DcR1* gene primer sequence

***Primer***	***Sequence (5’ – 3’)***	***X°C***	**P**
*DcR1-MF*	TTACGCGTACGAATTTAGTTAAC	50	127
*DcR1-MR*	CATCAAACGACCGAAACG		
*DcR1-UF*	GAATTTTTTTATGTGTATGAATTTAGT	55	136
*DcR1-UR*	CCATCAAACAACCAAAACA		

Note: CpG islands were bold and underlined; X°C: primer-annealing temperature. M: methylated, U: Unmethylated; F: Forward; R: Reverse; P: product size.

MSP assay was carried out in a total of 15 μl containing 3 μl bisulfite-modified template DNA, 0.75 unit iTaq DNA polymerase (Biorad), 0.5 μM each primer, 7.5 μl MyTaqTM Mix (Bioline). Thermal cycling was initiated at 95 °C for 5 min, followed by 40 cycles of denaturation at 95 °C for 30 sec, annealing at the X°C for 30 sec, extension at 72 °C for 30 sec, and a final extension at 72 °C for 10 min (Note: X°C was the specific annealing temperature for each specific methylated or unmethylated primer). The PCR products were run on 2% agarose gel with visualized by ethidium bromide staining. Then, MSP products were sequenced to confirm the specificity of primers, examine the efficiency of bisulfite modification and the hypermethylation status of target gene.

### Statistical analysis

Statistical analyses were performed by Medcalc® Version 12.7.0.0. The average frequency of methylation was calculated. The association between hypermethylation of *DcR1* and HPV infected status were examined by using Chi-square test. The differences in methylation frequencies of *DcR1*. Moreover, the association between hypermethylation of *DcR1* and risk of cervical cancer was estimated by computing OR, RR and 95% confidence intervals (CI).

## Results

### Hypermethylation profile of DcR1 gene promoter

**Fig. 1:**
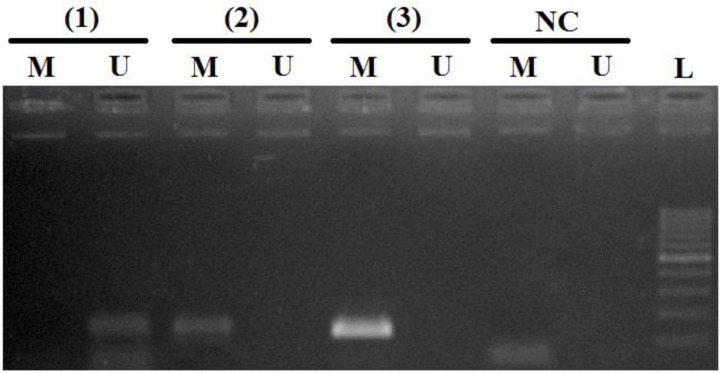
Methylated promoter of *DcR1* gene was analyzed on some clinical samples by MSP. (The MSP product was 127/136 bp in length. (1) non-HPV infected sample; (2) (3) high-risk HPV infected sample; NC: negative control; L: 100 bps ladder.

**Fig. 2:**
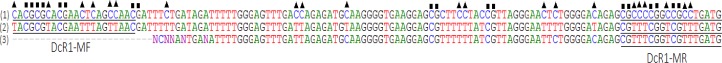
Sequencing profile of segment methylated of *DcR1* gene promoter. CG sites were marked as square symbols; Cytosine did not depend on the CpG site were marked as triangel symbols. (1) DNA sequence was without bisulfite modified; (2) DNA sequence was bisulfite modified; (3) The DcR1-MR sequencing by using the DcR1-MR.

The frequency of the promoter methylation of *DcR1* in clinical samples was evaluated by MSP method. Overall, the promoter frequencies for *DcR1* in high-risk HPV, low-risk HPV, and non-HPV infected samples were 50.0% (18 of 36 samples), 16.0% (4 of 25 samples), 14.6% (7 of 48 samples), respectively. Conversely, the promoter unmethylation frequencies were 50.0% (18 of 36 samples), 84.0% (21 of 25 samples) and 85.4% (41 of 48 samples) in high-risk HPV, low-risk HPV, and non-HPV infected samples, respectively. Methylation of *DcR1* in high-risk HPV infected samples was found to be significantly higher than other two groups (*P*=0.0005). The MSP products of samples hypermethylation and/or unmethylation in the promoter of *DcR1* were observed in electrophoresis with visualized by ethidium bromide staining and showed in Fig. 1. The MSP products of *DcR1* in clinical samples were observed in the band of 127 bps and 136 bps length in case of methylation and unmethylation, respectively. The sequencing of samples hypermethylated promoter region of representative sample revealed a conversion of unmethylated Cytosine, but not methylated Cytosine (Fig. 2). By sequencing, comparison between the nonbisulfite modified (Fig. 2(1)) and bisulfite modified (Fig. 2(2)), all methylated Cytosines were unchanged, marked as square symbols. Otherwise, all the unmethylated Cytosines, marked as triangle symbols, were changed into Thymine in bisulfite sequence. Additionally, three methylated CpG sites were observed in methylated reverse primer, which was according to the primer designed.

### Odds ratio, relative risk for promoter hyper-methylation in DcR1 gene

The odds ratio and relative risk values were computed between high-risk HPV infected group and low-risk HPV group combined with non-HPV infected group. The results show that odds ratio, relative risk values were 5.63 (95%CI=2.25–14.07, *P*<0.01) and 3.31 (95%CI=1.75–6.26, *P*<0.01), respectively.

## Discussion

Cervical cancer is the most commonly detected cancer and the main reason of mortality from cancer among females worldwide, particularly in Vietnam with the high prevalence of cervical cancer cases. The act of finding specific, predictive and prognostic biomarkers for cancer, considered as leading to increasing the survival rate, has been focusing and developing. Recently, DNA hypermethylation has been reported that it is a frequent mechanism plays an important criterion in many human cancers, such as breast cancer, cervical cancer, etc. by reducing the tumor repressor gene expression (11, 14–16). We examined the methylation status of *DcR1* gene promoter in liquid-based Papanicolaou (Pap) test sample by MSP method. Ideally, liquid-based Papanicolaou (Pap) sample, a non-invasive sample, contains exfoliating cells from the transformation zone of the cervix, thus, the presence of DNA in exfoliating cells has offered an opportunity to find out the predictive and prognostic biomarkers for cervical cancer (17–19). Therefore, Pap samples were used to analyze the hyper-methylation of candidate in Vietnamese cervical cancers. Additionally, the MSP method could rapidly assess the methylation status of virtually CpG sites, independent of the use of methylation-sensitive restriction enzymes (20). In methylation analysis assay, MSP method has been reported to be sensitive, which reached to 0.1% methylated alleles of given CpG island locus, and small quantities of DNA required, thus easily applied on non-invasive samples (12, 13, 20)

Up to now, there is limit research related to the evaluation of *DcR1* gene promoter methylation in cervical cancer, as well as whether or not an association between DNA methylation status and high-risk HPV infection, led to risk of cervical cancer. In current study, hypermethylation of the *DcR1* gene promoter was found in high frequency in high-risk HPV infected samples, counting for 50.0% (18 of 36 samples), compared to low-risk HPV infected samples, counting for 16.0% (4 of 25 samples) and non-HPV infected samples, counting for 14.6% (7 of 48 samples). The hypermethylation of *DcR1* gene was strongly associated to the HPV infection, particularly, high-risk HPV type infected, here, the infection of HPV 16 and/or 18 high-risk genotype infection were mentioned (*P*=0.0005). The viral infection has been already proven that it correlated to the epigenetic alteration, especially, the DNA hyper-methylation, in human cervical cancer. The high-risk HPV infection, such as type 16 and 18 infections, is the common cause of cervical cancer development. Additionally, the epigenetic factors, such as hypermethylation, have been suggested as contributing mechanisms to cervical tumorigenesis (3, 5, 10, 17, 21). Moreover, the virally encoded oncoproteins, such as HPV16 E7 associated in vitro and in vivo with the DNA methyltransferase 1 (DNMT1) considered as the enzymatic machinery involved in gene methylation.

DNA methyltransferase activity was upregulated by the binding of HPV-16 E7 and DNMT1, thus, increased the hypermethylation in many TSGs led to the development of human cancer of cervix (22). *DcR1* gene has been postulated to function as TSGs, in which the aberrant methylation in the promoter of *DcR1* has been reported in many human cancers, such as neuroblastoma tissue, prostate cancer, etc. (23, 24). Notably, no more data about *DcR1* promoter methylation in cervical cancer. Thus, high-risk HPV infection could promote the silencing of *DcR1* gene, contributing to development of cervical cancer. However, the mechanism of the down-regulation or non-expression of *DcR1* in tumorigenesis is not clear (23).

The hypermethylation of *DcR1* in cervical patients was significantly associated with an approximately 5.63-fold increase in the high-risk HPV infected group than compared to low-risk and/or non-HPV infected group (OR=5.63, 95%CI=2.25–14.07, *P*<0.01). Concerning to RR value, high-risk HPV infection significantly increased 3.31 times in the risk of *DcR1* hyper-methylation, leading to the inactivation of *DcR1* (RR=3.31, 95%CI=1.75–6.26, *P*<0.01).

Therefore, due to our data, identification of HPV high-risk genotype, HPV 16 and HPV 18 geno-type mentioned, and the hypermethylation of *DcR1* gene promoter were significant association and contribution to the risk and development of cervical cancer. Moreover, MSP method for *DcR1* hypermethylation detection and HPV typing in Pap test samples, non-invasive clinical samples, could be considered as the promising candidate, non-invasive biomarkers that could be potentially used for diagnosis and prognostic purposes in Vietnam.

## Conclusion

The promoter hypermethylation of *DcR1* was commonly found in the high-risk HPV genotype infection, counting for 50.0%, compared to low high-risk HPV infection or non-viral infection. Additionally, the significant association between *DcR1* gene hypermethylation and HPV exposure as well as the odds ratio and relative risk were found in the significant correlation, counting for 5.63 and 3.31, respectively were reported. In addition, the screening, which based on the detection of *DcR1* promoter hypermethylation, which combined the HPV genotyping, on non-invasive method, will offer the potential method for predictive and prognostic biomarkers for human cervical cancer in Vietnamese population. In further study, the present findings require extension to a larger number of samples and series on many potential genes in order to get the profile of methylated genes related to cancer of cervix.

## Ethical considerations

Ethical issues (Including plagiarism, informed consent, misconduct, data fabrication and/or falsification, double publication and/or submission, redundancy, etc.) have been completely observed by the authors.
